# A follow‐up study of a Chinese family with Waardenburg syndrome type II caused by a truncating mutation of *MITF* gene

**DOI:** 10.1002/mgg3.1520

**Published:** 2020-10-12

**Authors:** Shuzhi Yang, Cuicui Wang, Chengyong Zhou, DongYang Kang, Xin Zhang, Huijun Yuan

**Affiliations:** ^1^ Department of Otolaryngology, The 4th Medical Center Chinese PLA General Hospital Beijing China; ^2^ Department of Otorhinolaryngology Head and Neck Surgery Chinese PLA General Hospital Beijing China; ^3^ National Clinical Research Center for Otorhinolaryngologic Disease Chinese PLA General Hospital Beijing China; ^4^ Center for Medical Genetics Southwest Hospital Army Medical University Chongqing China; ^5^ Institute Of Otolaryngology Chinese PLA General Hospital Beijing China

**Keywords:** gene mutation, hearing loss, *MITF*, Waardenburg syndrome

## Abstract

**Background:**

Waardenburg syndrome (WS) is a highly clinically and genetically heterogeneous disease. The core disease phenotypes of WS are sensorineuronal hearing loss and pigmentary disturbance, which are usually caused by the absence of neural crest cell‐derived melanocytes. At present, four subtypes of WS have been defined, which are caused by seven genes. Waardenburg syndrome type 2 (WS2) is one of the most common forms. Two genes, *MITF* and *SOX10*, have been found to be responsible for majority of WS2.

**Methods:**

In this study, we performed a clinical longitudinal follow‐up and mutation screening for a Chinese family with Waardenburg syndrome type II.

**Results:**

A diversity of clinical manifestations was observed in this WS2 family. In addition to the congenital hearing loss of most affected family members, progressive hearing loss was also found in some WS2 patients. A nonsense mutation of c.328C>T (p.R110X) in *MITF* was identified in all affected family members. This mutation results in a truncated MITF protein, which is considered to be a disease‐causing mutation.

**Conclusion:**

These findings offer a better understanding of the spectrum of *MITF* mutations and highlight the necessity of continuous hearing assessment in WS patients.

## Graphical abstract

The present study describes a large Chinese WS2 family with a highly clinical heterogeneity. Clinical longitudinal follow‐up revealed progressive hearing loss in some patients. A nonsense mutation of c.328C>T (p.R110X) in *MITF* was identified.

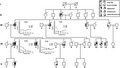

## 1 INTRODUCTION

Waardenburg syndrome (WS) is an autosomal dominant genetic disorder with incomplete penetrance. Primary symptoms of WS include sensorineural hearing loss (SNHL), hypopigmentation of the iris, hair, and skin (e.g., heterochromia iridum, white forelock, and patchy hypopigmented skin (Read & Newton, 1997). Four types of WS have been classified based on the clinical manifestations. WS type 1 (WS1, OMIM #193500) and type 2 (WS2, OMIM #193510) have very similar features but are distinguished by dystopia canthorum, which is only present in WS1. WS type 3 (WS3, OMIM #148820) is characterized by dystopia canthorum and upper limb abnormalities. WS type 4 (WS4, Shah‐Waardenburg syndrome, OMIM #277580), has the additional feature of Hirschsprung disease, in which patients may have anomaly of the enteric nervous system and suffer from functional colonic obstruction. Among these types, WS1 and WS2 are the most common forms of WS.

Highly phenotypic heterogeneity was observed in WS2, even in the same family. SNHL can occur either bilaterally or unilaterally, and heterochromia iridum manifests as very pale blue eyes, different colored eyes, or an eye with segments of two different colors. Other less‐common clinical signs include abnormal pigmentation disturbances, such as white forelock, early graying, and hypo‐ or hyperpigmented skin patches (Tassabehji et al., 1995). Consistent to the diversity of clinical manifestation, WS2 is genetically heterogeneous. At present, five genes, *MITF*, *SOX10*, *SNAI2*, *EDNRB*, and *KITLG*, have been associated with WS2 (Bondurand et al., 2007; Pingault et al., 2010; Sanchez‐Martin et al., 2002; Zazo Seco et al., 2015). However, the link between the causative genes and specific phenotypes is not completely clear. It has been proposed that the *MITF* gene is responsible for approximately 27.6% of WS2 cases and the *SOX10* gene for approximately another 4.6% (Song et al., 2016). *SNAI2*, *EDNRB*, and *KITLG* are only described in sporadic cases, indicating that they are not major causes of WS2. To date, more than 54 disease‐causing mutations have been identified in *MITF* gene in patients with WS2 or Tietz syndrome. Among these mutations, at least 22 are identified in Chinese WS2 patients ("The Human Gene Mutation Database at the Institute of Medical Genetics in Cardiff," 2019 Oct 13), suggesting that the *MITF* gene has a high mutation frequency in Chinese WS2 patients.

Hearing loss (HL) is the main complaint of WS2 patients. A meta‐analysis of HL in Waardenburg syndrome revealed that in the WS2 patients with *MITF* defects, progressive HL was not rare (Song et al., 2016). Here, we performed a clinical longitudinal follow‐up and mutation screening for a large Chinese WS2 family. Highly clinical diversities of deafness and heterochromia iridum were observed in this family, some patients manifested progressive hearing loss. A nonsense *MITF* gene mutation was identified. *MITF* gene mutation‐positive family members showed a little lower penetrance of HL (61.5%, 8/13 cases), 4 out of 13 cases maintained normal hearing during 13 years follow‐up period.

## 2 MATERIALS AND METHODS

### 2.1 Subjects

**FIGURE 1:**
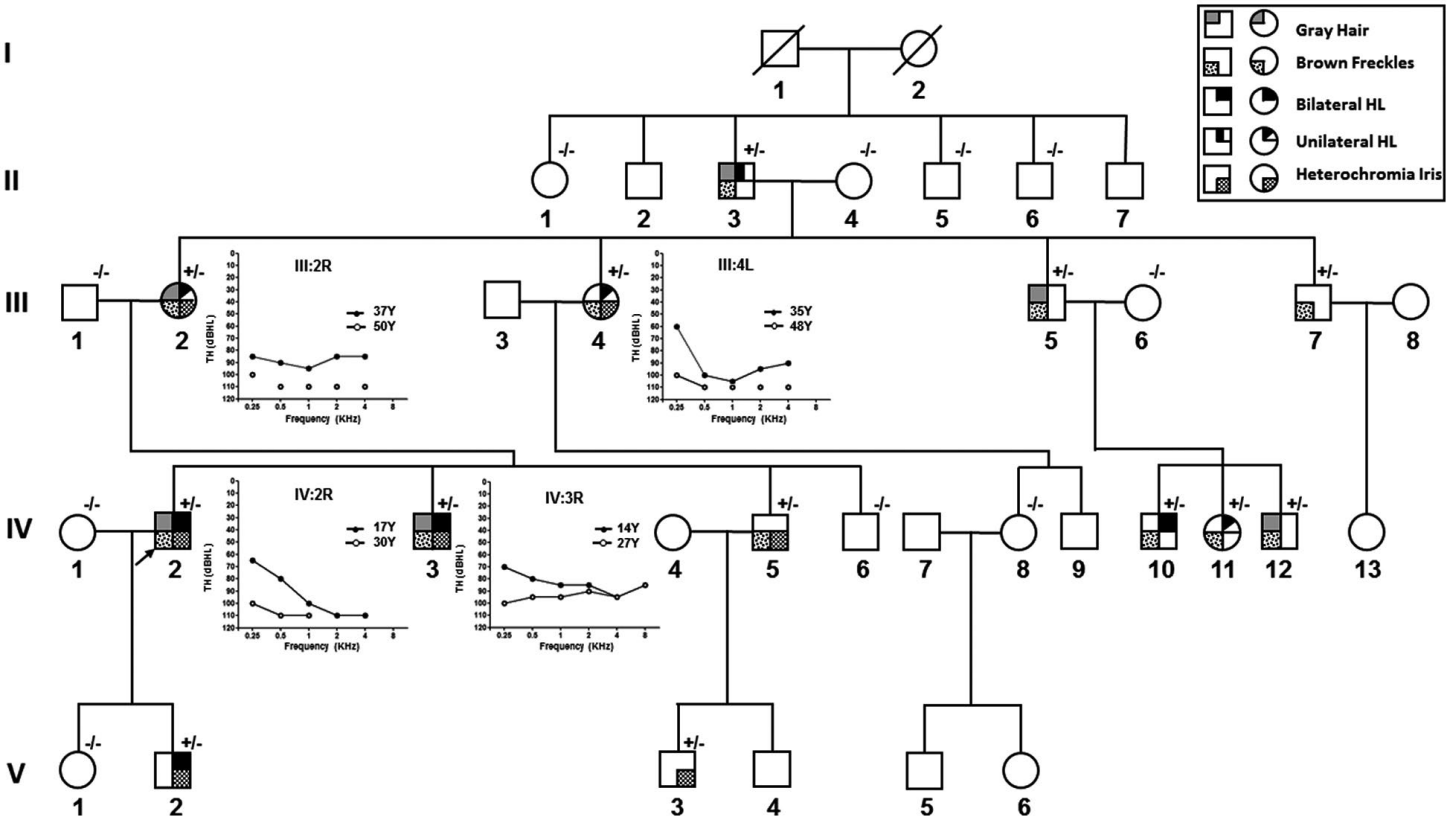
Pedigree of family JX‐WS01 with WS2, and audiograms showing threshold shifts of individual III:2, III:4, IV:2, and IV:3. Progressive hearing loss was found in this family in the period of 13 years of follow‐up. Disconnected circle means that thresholds are undetectable even at the level of the maximal output of audiometer. Y=year; TH=threshold

A large WS2 family, named JX‐WS01, was identified in 2004 in southeastern China and was followed up for the next 13 years with the last visit in 2017 (Figure 1). This family included four generations at the initial visit and expanded to five generations at the last visit in 2017. In total, the five‐generation pedigree including 36 family members from the youngest four generations participated in this study. This study was approved by the Ethics Committee of the Chinese PLA General Hospital. Written consent was obtained from all adult patients and the guardians of child patients prior to the clinical evaluation and blood sample collection.

### 2.2 Clinical evaluation

Thirty‐four family members were examined in person, including comprehensive clinical history and neurotological, ophthalmological, dermatological, and audiological assessments. The ophthalmological examination included visual acuity measurements, visual field examination, and fundus ophthalmoscopy. Special attention was given to the color of skin, hair, and iris as well as developmental defects such as dystopia canthorum and limb abnormalities. All subjects received audiological assessments, which included otoscopy, tympanometry (OTOflex 100, MADSEN, Denmark), and pure‐tone audiometry (ITERA, MADSEN, Denmark). For young patients with age less than 3, auditory brain‐stem response (ABR) was measured (ICS chartr EP 200, MADSEN, Denmark) because these individuals were unable to cooperate with the pure‐tone audiometry test. The degree of hearing loss was defined based on the pure‐tone average (PTA), which is the average of the thresholds at 500, 1000, and 2000 Hz, with follow levels: normal, <26 dB HL; mild, 26–40 dB HL; moderate, 41–70 dB HL; severe, 71–90 dB HL; and profound, >90 dB HL.

### 2.3 Blood sample collection and DNA extraction

Among the 34 family members, 23 provided blood samples for genotyping. DNA was extracted from the leukocytes of blood samples using a DNA extraction kit (Huasuo Co. Ltd, Shanghai, China). Two hundred DNA samples from unaffected individuals with Chinese genetic background comprised the control genomic DNA samples.

### 2.4 Sanger sequencing

Direct sequencing was performed for mutation screening. All coding exons and 200 bp of the flanking intron splicing sites of the WS2‐related hot genes, *MITF* and *SOX10* were amplified by polymerase chain reaction (PCR) using the primers as described before (S. Yang et al., 2013). All PCR amplifications were carried out using 40 ng of genomic DNA and 2 pmol of each primer. PCR cycles included 94℃ for 4 min, then 30 cycles of denaturation at 94℃ for 30 s, annealing at various temperature for 30 s for the different primers, and extension at 72℃ for 30 s, followed by a 7‐min final extension at 72℃. PCR products were ethanol‐purified and sequenced in both directions using the ABI BigDye Terminator Cycle Sequencing Kit (ver. 3.1; ABI Applied Biosystems, Foster City, CA), with the same primers used for PCR. The raw sequence data produced by the ABI_Prism 3100 DNA sequencer were aligned with the wild‐type sequence using the Chromas LITE Version 2.1 program.

## 3 RESULTS

### 3.1 Clinical manifestation

Initial visit was carried out in 2004. The proband (IV:2) was a 17‐year‐old boy with bilateral profound hearing loss and bilateral brilliant blue irides at birth. Brown freckles gradually appeared on his face when he was 5–6 years old, and his hair started to turn gray at the age of 14. Table 1 summarized the main clinical findings of 13 affected family members of JX‐WS01.

**TABLE 1:** Summary of Clinical Data for 13 family members of JX‐WS01 who carried the *MITF* gene mutation

No.	Gender	Age of first visit (Years)	HL at Onset (years)	Iris	Skin	G	W	Severity of HL	
								Left ear	Right ear
II‐3	Male	60	32	−	+	+	1.90	Normal	Profound
III‐2	Female	37	33	C	+	+	1.60	Normal	Severy
III‐4	Female	35	33	B	+	−	1.60	Profound	Normal
III‐5	Male	32	−	−	+	+	1.61	Normal	Normal
III‐7	Male	27	−	−	+	−	1.78	Normal	Normal
IV‐2	Male	17	Prelingual	C	+	+	1.93	Profound	Profound
IV‐3	Male	14	Prelingual	B	+	+	1.60	Profound	Severy
IV‐5	Male	13	−	A	+	−	1.93	Normal	Normal
IV‐10	Male	12	Prelingual	−	+	−	1.71	Profound	Profound
IV‐11	Female	8	15	−	+	−	1.76	Normal	Profound
IV‐12	Male	5	−	−	+	+	1.73	Normal	Normal
V‐2	Male	1.5	Prelingual	C	−	−	2.28	Profound	Profound
V‐3	Male	3.5	−	A	−	−	2.10	Normal	Normal

Abbreviations: −, sign absent; +, sign present; A, complete heterochromia iridis; B, partial or segmental heterochromia iridis; C, brilliant blue iris; G, early gray hair; HL, hearing loss; Skin, numerous brown freckles on the face; W, W index.

Hyperpigmentation of skin in the style of numerously brown freckles on the face was a major phenotype of WS2 in this family. Eleven of 13 affected family members manifested this character under teenager (11/13, 84.6%). Premature graying hair was another abnormality of pigmentary distribution observed in this pedigree with 6 cases affected (6/13, 46.2%).

HL was the second common phenotype of WS2 in this family. Eight patients among 13 affected family members (61.5%) showed the signs of HL with different profiles, ranging from unilateral (4/8, 50%) to bilateral (4/8, 50%), from congenital to postlingual, and from moderate to profound hearing loss. Intriguingly, the unilateral, late‐onset SNHL was observed primarily in the second and third generations. In contrast, bilateral, congenital deafness was more prominently found in the fourth and fifth generations.

**FIGURE 2:**
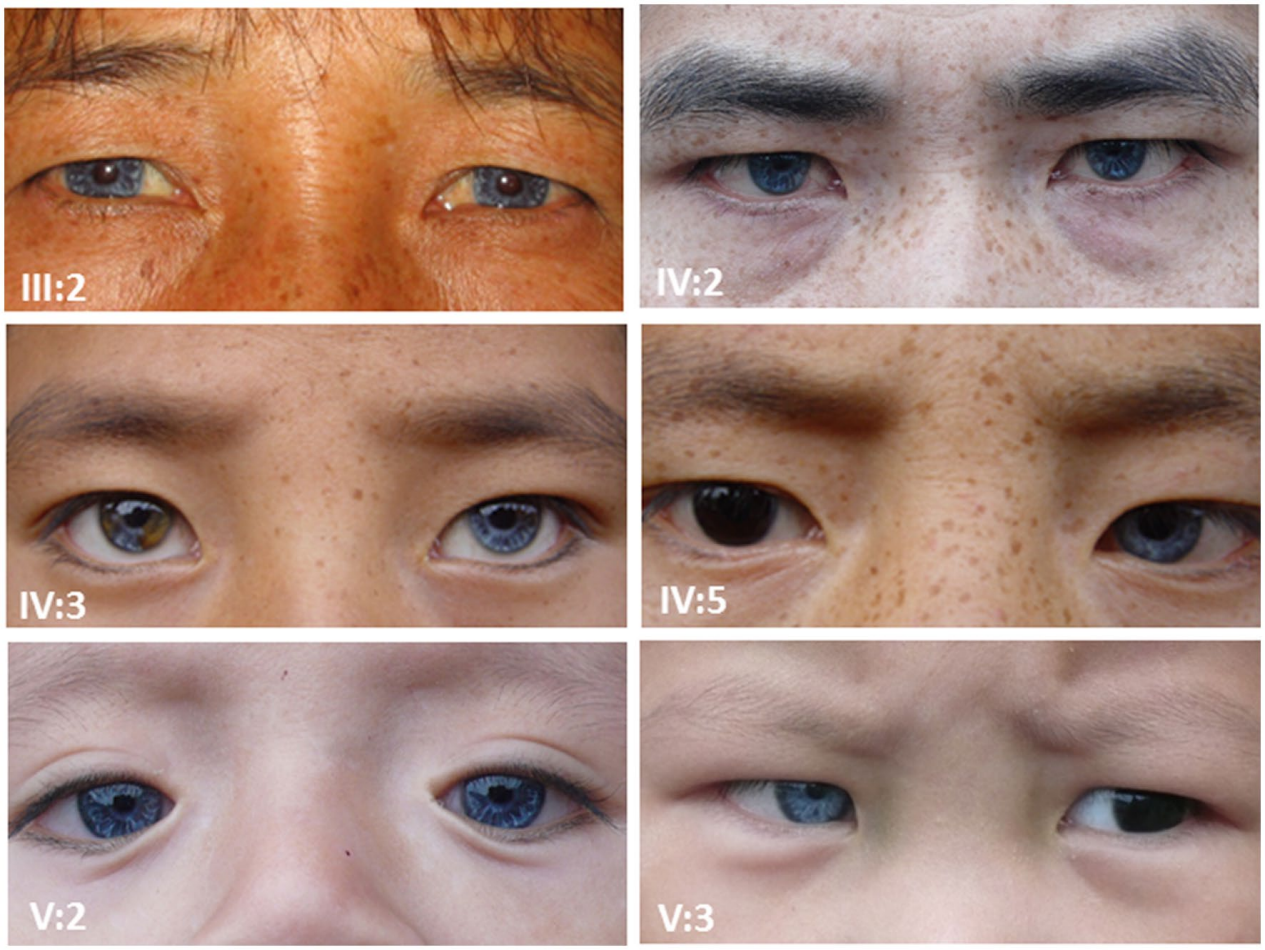
Diverse heterochromia iris in family JX‐WS01. III:2, IV:2, and V:2: brilliant blue iris; IV:3: partial heterochromia iridis; IV:5 and V:3: complete heterochromia iridis

Various types of heterochromia iris were the third common features observed in this WS2 family (Figure 2). Among the seven affected individuals who manifested heterochromia iridis, three were characterized brilliant blue iris, two were bilateral partial heterochromia, and the remaining two were complete heterochromia iridis. No visual problem was found.

The average W index of all the 13 affected individuals was 1.81, less than 1.9, Meeting the clinical diagnostic criteria of WS2 proposed by the WS consortium (Farrer et al., 1992).

### 3.2 Mutational analysis

**FIGURE 3:**
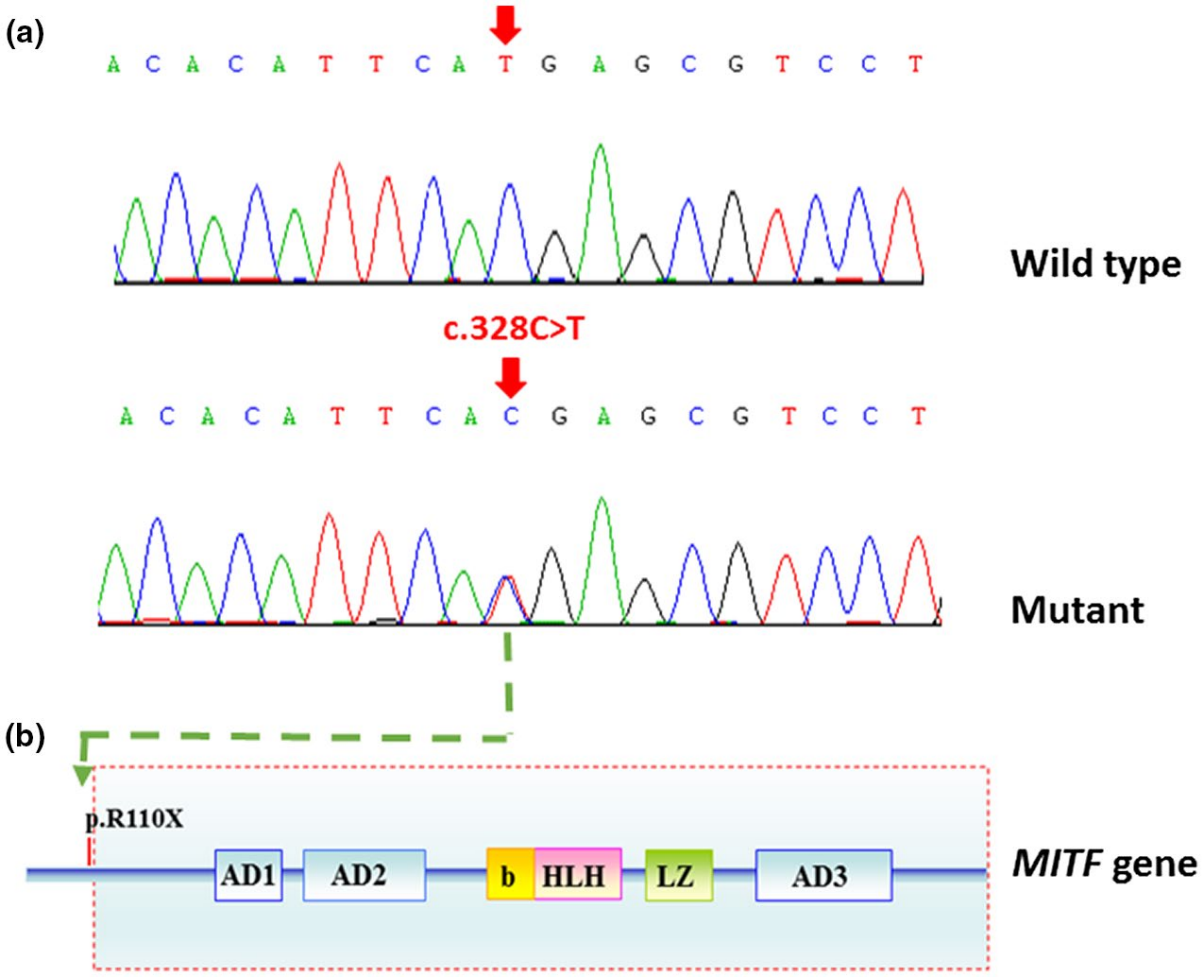
Mutational analysis of affected members of family JX‐WS01 and healthy controls. (a) DNA sequence chromatograms showing a heterozygous nonsense mutation of c.328C>T in *MITF* in patients. This mutation is absent in wild‐type controls. The mutation alters the 110th codon which encodes arginine into a premature termination codon (p.R110X). (b) The schematic illustration of MITF protein. Mutation p.R110X causes MITF to loss three‐quarters of the length of protein sequence, including a basic DNA binding domain, helix‐loop‐helix motif, and a leucine zipper. AD1‐3, transactivation domains; b, basic DNA binding domain; HLH, helix‐loop‐helix motif; LZ, leucine zippedomain

Mutational screening showed that a single heterozygous C to T mutation at nucleotide position 328 of *MITF* gene (c.328C>T, Figure 3a). This mutation altered the 110th arginine to a premature termination codon (p.R110X). As a result, the MITF protein lost about ¾ of its length of series, leading to the loss of important structures, including a basic DNA binding domain, a helix‐loop‐helix motif and a leucine zipper (Figure 3b). Mutation p.R110X was detected in all of other 12 affected family members. Meanwhile, unaffected family members or 200 unrelated normal controls was negative for this mutation.

### 3.3 Clinical Follow‐up

Second visit was carried out in 2017. This family expanded from four generations to five generations with added two new affected family members. A total of 11 patients who had *MITF* gene mutation were re‐examined by hearing test. Four subjects (III:5, III:7, IV:5, and IV:12) had maintained normal hearing in the past 13 years, and four subjects (III:2, III:4, IV:2, and IV:3) with hearing loss manifested a little progressive with average threshold shift ranging from 15.0 to 21.7 dB (Detailed hearing shift See Figure 1).

## 4 DISCUSSION

WS2 is a well‐defined, phenotypically heterogeneous disease. Diverse phenotypes and incomplete penetrance can be seen in a single family. Here, we report a large Chinese family with WS2. This pedigree displays diverse clinical manifestations. HL is the main symptom the patients concerned in this family, the penetrance of HL observed in this pedigree is 61.5%, slightly lower than that reported in a previous meta‐analysis, which summarized the findings from 417 WS patients reported in 73 articles (Song et al., 2016). SNHL is found in about 90% of 115 patients with WS2 that had *MITF* gene mutation. Among these patients, 60% displayed severe to profound hearing loss, 89.5% displayed bilateral hearing loss, and 10.5% had unilateral hearing loss. While the cause of the lower penetrance of HL is not clear, genetic modification, environmental factors, and our small sample size might contribute to this difference.

A thorough literature review revealed that few studies followed WS patients in a longitudinal way. So the progression of HL in WS patients is unclear. In a cohort of 62 patients with audiometric data available, hearing loss were found stable in 57 patients versus progressive in 5 individuals (91.9% vs. 8.1%) (Song et al., 2016). In this study, we performed a longitudinal follow‐up for 13 years. We found that four patients with five ears displayed hearing deterioration with average threshold shift ranging from 15.0 to 21.7 dB. This finding reveals that the progressive hearing loss is not rare in WS patients, and emphasizes the need for continuous hearing evaluation for WS patients.

It is interesting that the unilateral, late‐onset SNHL was observed primarily in the second and third generations. In contrast, bilateral, congenital deafness was more prominently found in the fourth and fifth generations. The exact explanation is not clear, but the genetic anticipation might contribute for this phenomenon.

Brown freckles on the face is the most common pigmentary abnormalities, which was found in 11 individuals out of 13 patients (84.6%). This penetrance is slightly higher than another two Chinese WS2 cohort, (7/10, 70%) and (5/20, 25%), reported before (L. Sun et al., 2016; S. Yang, et al., 2013). Noticeably, brown freckles often appeared slowly at the age 4–12. For example, IV:12 did not show any signs of WS2 at the age 5 when we first checked him in 2004. However, he developed abnormal pigmentation disturbances on his face and hair at the time we checked him again in 2017. Unlike patchy, depigmented skin that is more common in Western cases, brown freckles on the skin is a common phenotype of skin pigmentary abnormalities in Chinese WS2 patients.

Three types of heterochromia iridum, including brilliant blue iris, partial heterochromia, and complete heterochromia iridis, were observed in this WS2 family. Compared with the usual dark brown iris in Asian population, distinct blue iris makes it easier to be identified at birth. In contrast to late identifying of HL, heterochromia iridum may imply an early diagnosis of WS.

A nonsense *MITF* gene mutation was identified in this family. Mutation c.328C>T (p.R110X) co‐segregated with disease in this family, all the 13 affected family members carried this defect. This mutation is predicted to result in a truncated MITF protein, leading to loss of about ¾ length of series including important structures, such as a basic DNA binding domain, HLH motif and a ZIP, and thought to have pathogenicity. This mutation was confirmed in other two unrelated sporadic WS2 patients. One is Caucasian patient, he is 16 years old boy, who suffered from heterochromia iridis and mild hearing loss. Another one is Chinese patient, she is 15‐year‐old girl. She manifested heterochromia iridum, premature graying hair, excessive freckles, patchy skin depigmentation, and profound hearing loss (Sun et al., 2016; Wildhardt et al., 2013). Intriguingly, Hearing is normal in 5 of 13 affected family members even in the presence of this mutation, and 4 of 5 individuals have kept normal hearing in the past 13 years. The exact pathomechanism of this phenomena is not clear. Different from findings in animal models of WS2, *MITF* gene mutations in human are always in heterozygous status. Therefore the haploinsufficiency (half normal levels) of MITF could account for WS2 in a dosage‐dependent fashion (Tachibana, 1997).

It is estimated that approximately 27.6% of WS2 cases is caused by mutation of *MITF* gene (Song et al., 2016). The MITF protein belongs to the Myc superfamily of b‐HLH‐Zip (basic DNA binding domain, helix‐loop‐helix, and leucine‐zipper domain) proteins. MITF has roles in the differentiation and development of neural crest cells (NCCs), and in the survival, migration, differentiation, and development of melanocytes (Hemesath et al., 1994; Sauka‐Spengler & Bronner‐Fraser, 2006). It is well known that the target cells of MITF in cochlear is pigmentary cells of stria vascularis, which is essential for production of endolymphatic potential (Chen, et al., 2016). Findings found in animal models showed that early degeneration of the intermediate cells of the cochlear stria vascularis. Up to date, at least 54 *MITF* gene mutations have been identified in WS2 and Tietz syndrome ("The Human Gene Mutation Database at the Institute of Medical Genetics in Cardiff," 2019 Oct 13). Among these mutations, 22 are found exclusively in Chinese patients with WS2, accounting for about 41% of the *MITF* gene mutations listed in the database (Table 2).

**TABLE 2:** Summary of *MITF* Gene variants Identified in Chinese WS2 Patients

No.	Nucleotide change^a^	Amino acid change^b^	Variant class	References
1	c.20A>G*	p.Tyr7Cys	DM	Yang, et al. (2013))
2	c.332C>T*	p.Ala111Val	DM	Yang, et al. (2013))
3	c.328C>T*	p.Arg110X	DM	This study, (Sun et al., 2016; Wildhardt et al., 2013)
4	c.494delC*	p.P165fs	DM	Sun et al. (2016)
5	c.575delC*	P.Thr192LysfsX18	DM	Chen et al. (2010)
6	c.608G>A*	p.R203K	FP	Chen et al. (2010)
7	c.639delA*	p.Glu213AspfsX8	DM	Chen et al. (2008)
8	c.641G>A*	p.R214Q	DM	Sun et al. (2016)
9	c.647_649 del GAA*	p. Arg217del	DM	Yang, et al. (2013))
10	c.648_650delAAG*	p. Arg217del	DM	Chen et al. (2010)
11	c.649_651delAGA	p. Arg217del	DM	Chen, et al. (2016); Tassabehji et al. (1995)
12	c.649A>G*	p.Arg217Gly	DM	Yang, et al. (2013))
13	c.650G>T*	p.Arg217IIe	DM	Chen et al. (2010)
14	c.710+1G>T*	IVS7 ds G‐T+1/ p.P237fs	DM	Sun et al. (2016)
15	c.718C>G*	p.R240G	DM	Zhang et al. (2018)
16	c.742_747delAAAGCAinsTAG*	a truncated MITF protein with only 247 of the 419 wild‐type amino acids	DM	Yan et al. (2011)
17	c.763C>T*	p.Arg255X	DM	Sun et al. (2016), Yang, et al. (2013)), Yang, et al. (2013))
18	c.641_643delGAA*	p.Arg215del	DM	Shi et al. (2016)
19	c.808C>T*	p.R270X	DM	Sun et al. (2016)
20	c.1060C>A*	p.L354I	DM？	Wang et al. (2018)
21	c.1260G>C*	p.X420Y	DM	Sun et al. (2017)
22	c.1258T>C*	p.X420Qext51	DM	Sun et al. (2016)

Abbreviations: DM, disease‐causing mutation; DM?:, likely disease‐causing mutation; FP, In vitro or in vivo functional polymorphism.

*, the mutation has been firstly described in the Chinese population.

^a^ Description of the mutations is based on GenBank Reference Sequence for the M isoform of *MITF* gene: NM_000248.3.

^b^ Amino acid numbering is based on GenBank Reference Sequence: NP_000239.1.

In summary, we report detailed clinical findings in a longitudinal follow‐up study of a five‐generation Chinese family with WS2. We found progressive hearing loss occurred in some patients, which underscores the need for clinical follow‐up. Our molecular analysis provides the evidence for the occurrence of a nonsense mutation p.R110X of MITF. Further study should be focused on the molecular basis for these patients, who carried mutation p.R110X of MITF but still have normal hearing.

## CONFLICT OF INTEREST

The authors declare that they have no competing interests.

## AUTHOR CONTRIBUTIONS

SY, DK, and XZ: did all the experiments, and participate in the analysis and writing up of the manuscript. CW, CZ, and SY did the clinical assessment and recruitment of the patients and their family members. SY and HY designed the study, secured the funding, analyzed the data, and wrote up the manuscript. All authors read and approved the final manuscript.

## ETHICAL APPROVAL AND CONSENT TO PARTICIPATE

The entire procedure was approved by the Ethics Committee of the Chinese PLA General Hospital and carried out with written informed consent of the patients and the parents.
